# Supplementary material for: Defining Composition and Function of the Rhizosphere Microbiota of Barley Genotypes Exposed to Growth-Limiting Nitrogen Supplies
Source: mSystems. 2022 Nov 7;7(6):e00934-22. doi: 10.1128/msystems.00934-22 (PMC9765016; doi:10.1128/msystems.00934-22)
Supplement: TABLE S1 [file msystems.00934-22-s0009.docx]

Table S1. Composition of the nutrient solutions used in this study. The solution was applied with watering of the plants at a rate of 25 ml of the nutrient solution per Kg of soil.

|  | **N 100%** | **N 25%** | **N 0%** |
| --- | --- | --- | --- |
| 2(NH_4_) SO_4_ | 25mM | 6.25mM | 0 |
| Ca(NO_3_)_2_ | 40mM | 10mM | 0 |
| KNO_3_ | 10mM | 2.5mM | 0 |
| Mg SO_4_ | 3mM | 3mM | 3mM |
| FeEDTA + | 100μM | 100μM | 100μM |
| Micronutrients* | See below | | |
| KH_2_PO_4_ | 1mM | 1mM | 1mM |
| CaSO_4_ + 2H_2_O | 0 | 0.019mM | 0.025mM |
| CaCl_2_ | 0 | 0.06mM | 0.08mM |
| KCl | 0 | 0.19mM | 0.25mM |

*Micronutrients concentration: 6 μM MnCl_2_, 23 μM H_3_BO_3_, 1.6 μM CuSO_4_, 0.6 μM ZnSO_4_, 1 μM Na_2_MoO_4_, 1 μM CoCl_2_.
