# Supplementary figures and images for: Defining Composition and Function of the Rhizosphere Microbiota of Barley Genotypes Exposed to Growth-Limiting Nitrogen Supplies
Source: mSystems. 2022 Nov 7;7(6):e00934-22. doi: 10.1128/msystems.00934-22 (PMC9765016; doi:10.1128/msystems.00934-22)

**N0%**

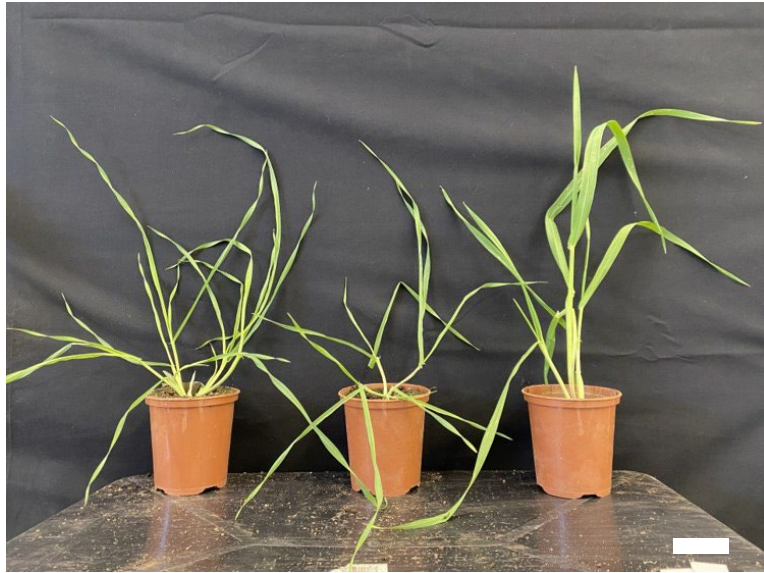

Desert

North

Elite

**N25%**

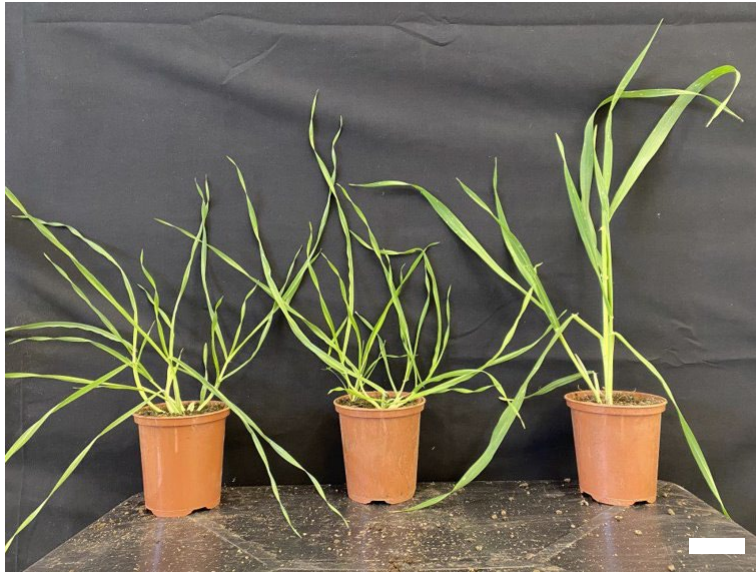

Desert

North

Elite

**N100%**

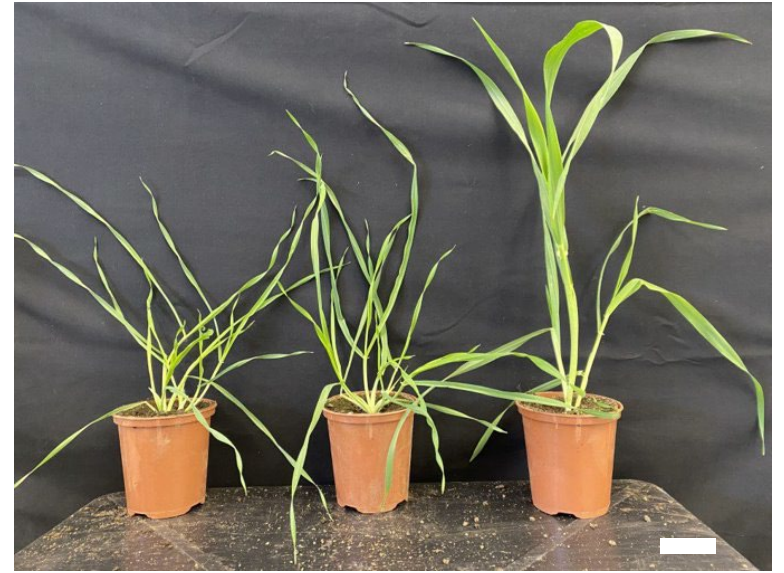

Desert

North

Elite

Supplement: FIG S1 [file msystems.00934-22-s0001.pdf]

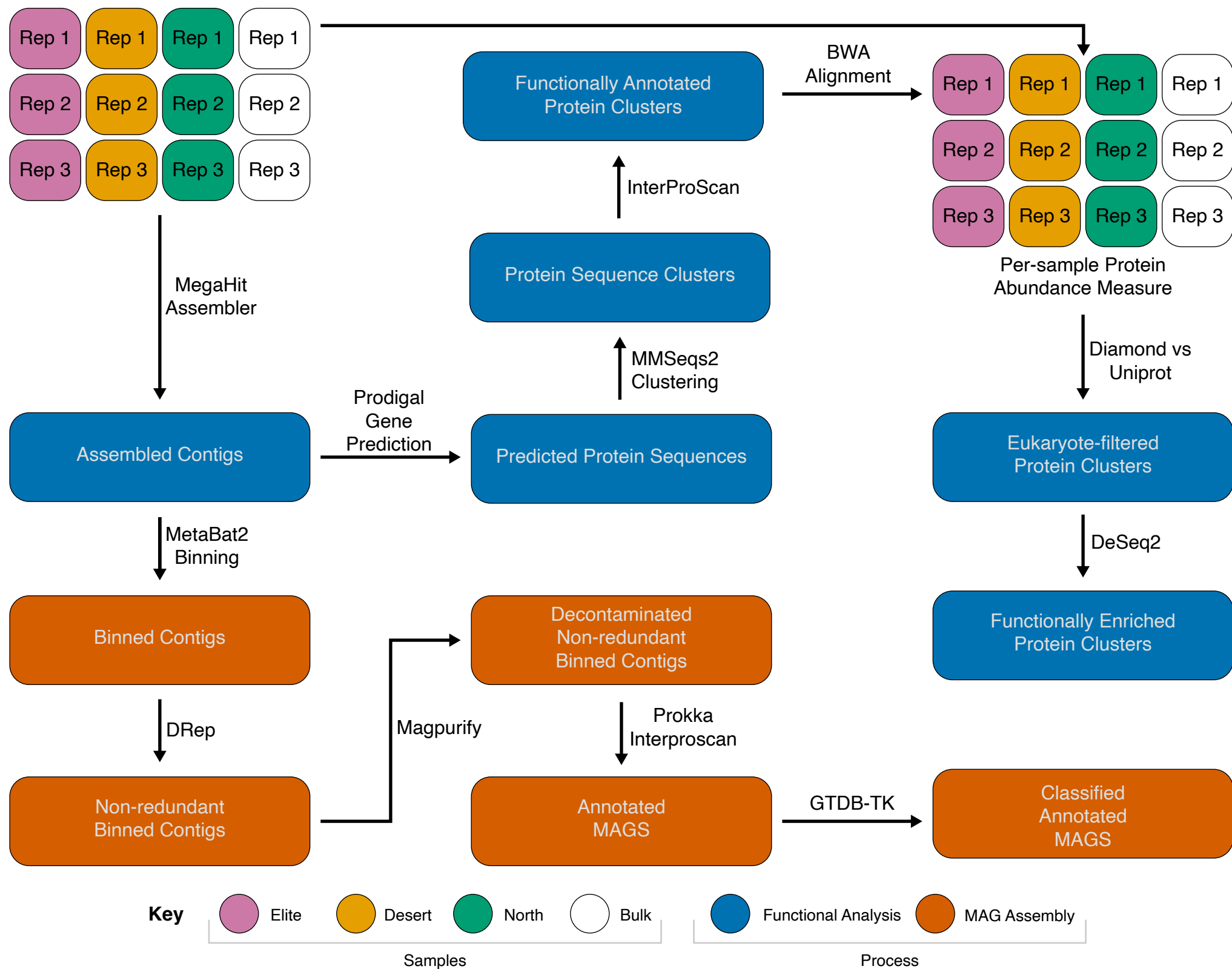

Supplement: FIG S2 [file msystems.00934-22-s0002.pdf]

Counts

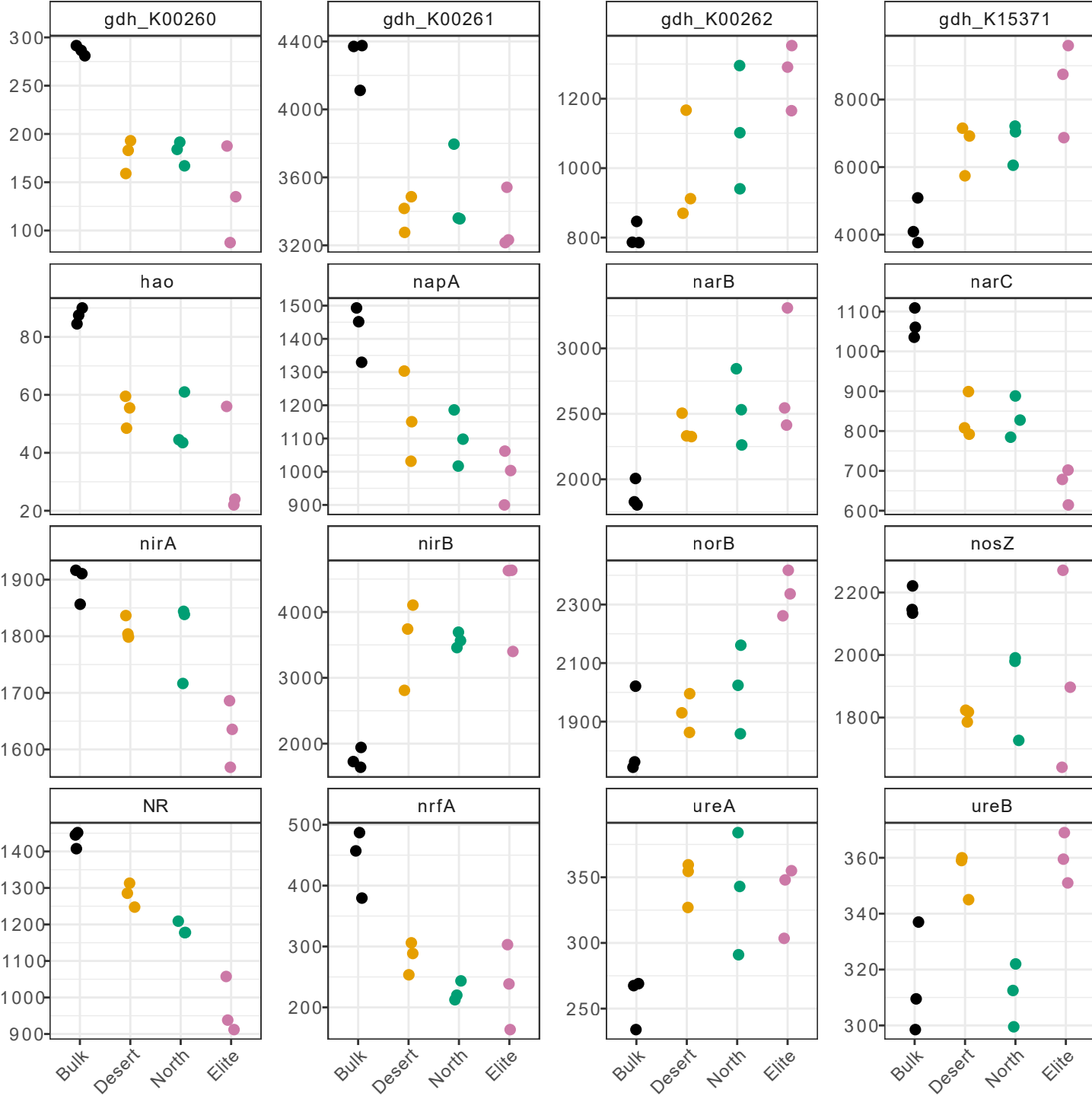

Supplement: FIG S3 [file msystems.00934-22-s0003.pdf]

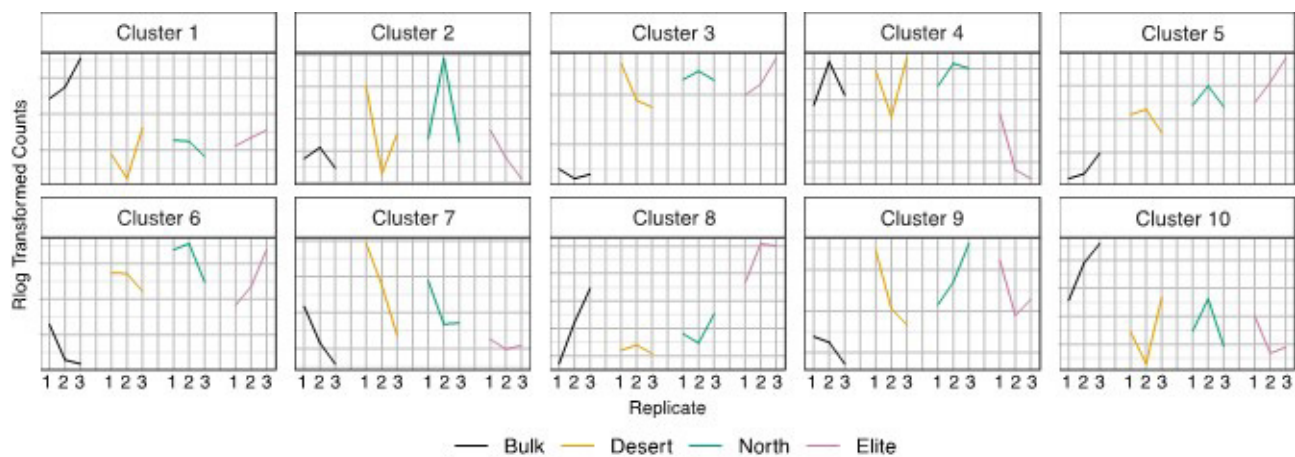

Supplement: FIG S4 [file msystems.00934-22-s0004.pdf]

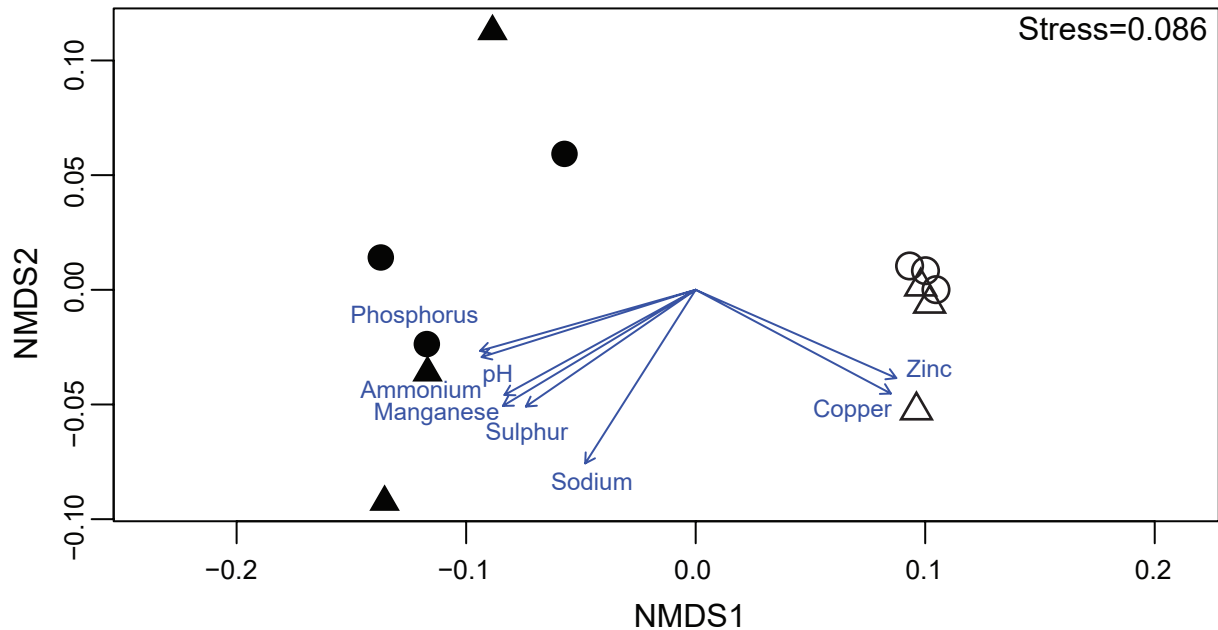

Genotype

○ Elite

△ Desert

Treatment

□ Native

■ Heat-treated

Supplement: FIG S6 [file msystems.00934-22-s0006.pdf]

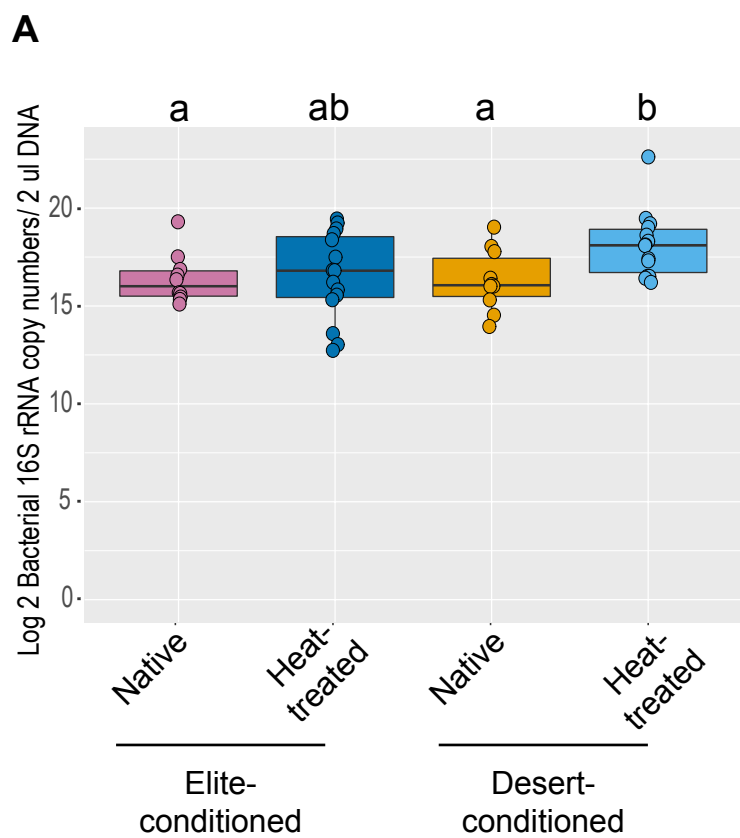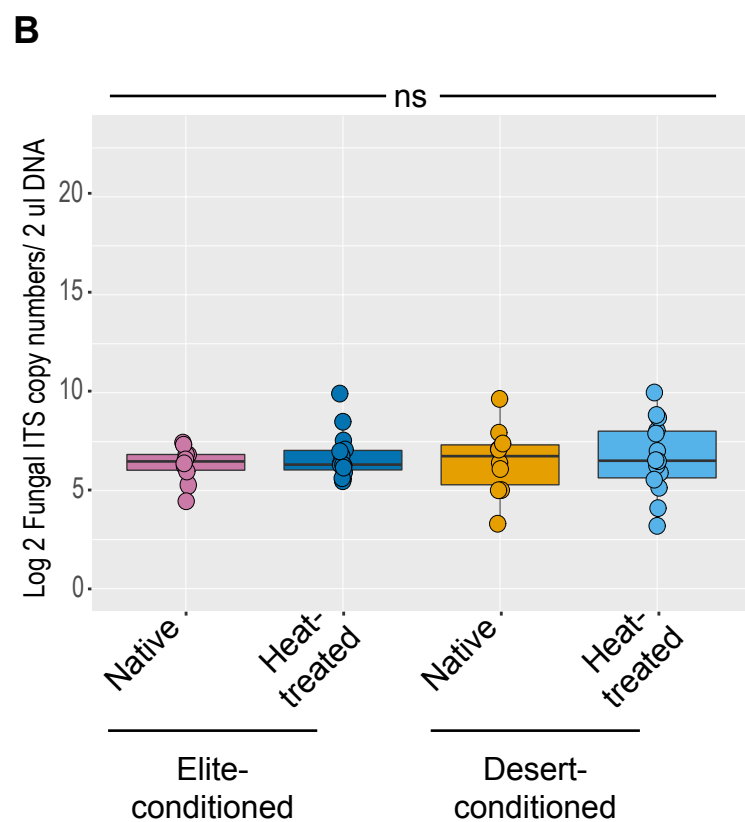

Supplement: FIG S7 [file msystems.00934-22-s0007.pdf]

**A**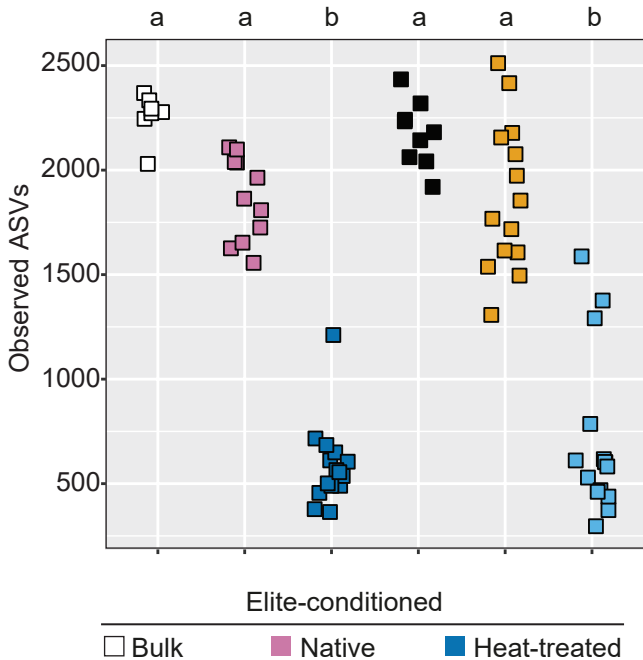**B**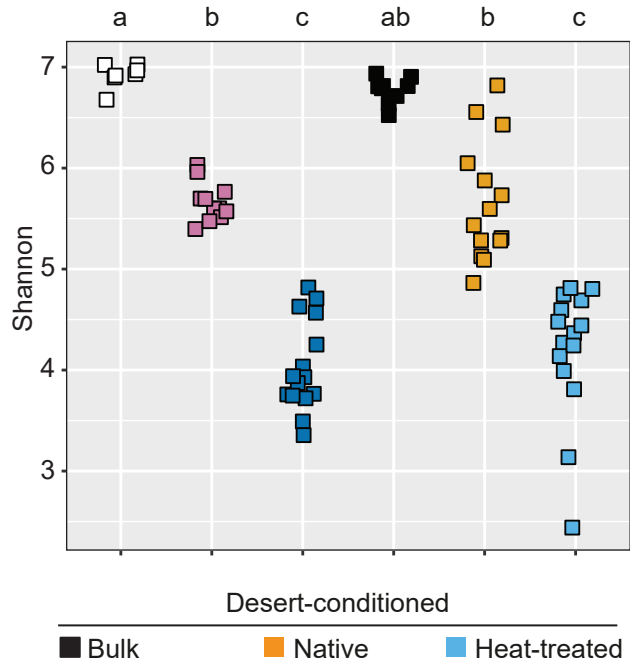

Supplement: FIG S8 [file msystems.00934-22-s0008.pdf]
