# Supplementary material for: Defining Composition and Function of the Rhizosphere Microbiota of Barley Genotypes Exposed to Growth-Limiting Nitrogen Supplies
Source: mSystems. 2022 Nov 7;7(6):e00934-22. doi: 10.1128/msystems.00934-22 (PMC9765016; doi:10.1128/msystems.00934-22)
Supplement: FIG S5 [file msystems.00934-22-s0005.pdf]

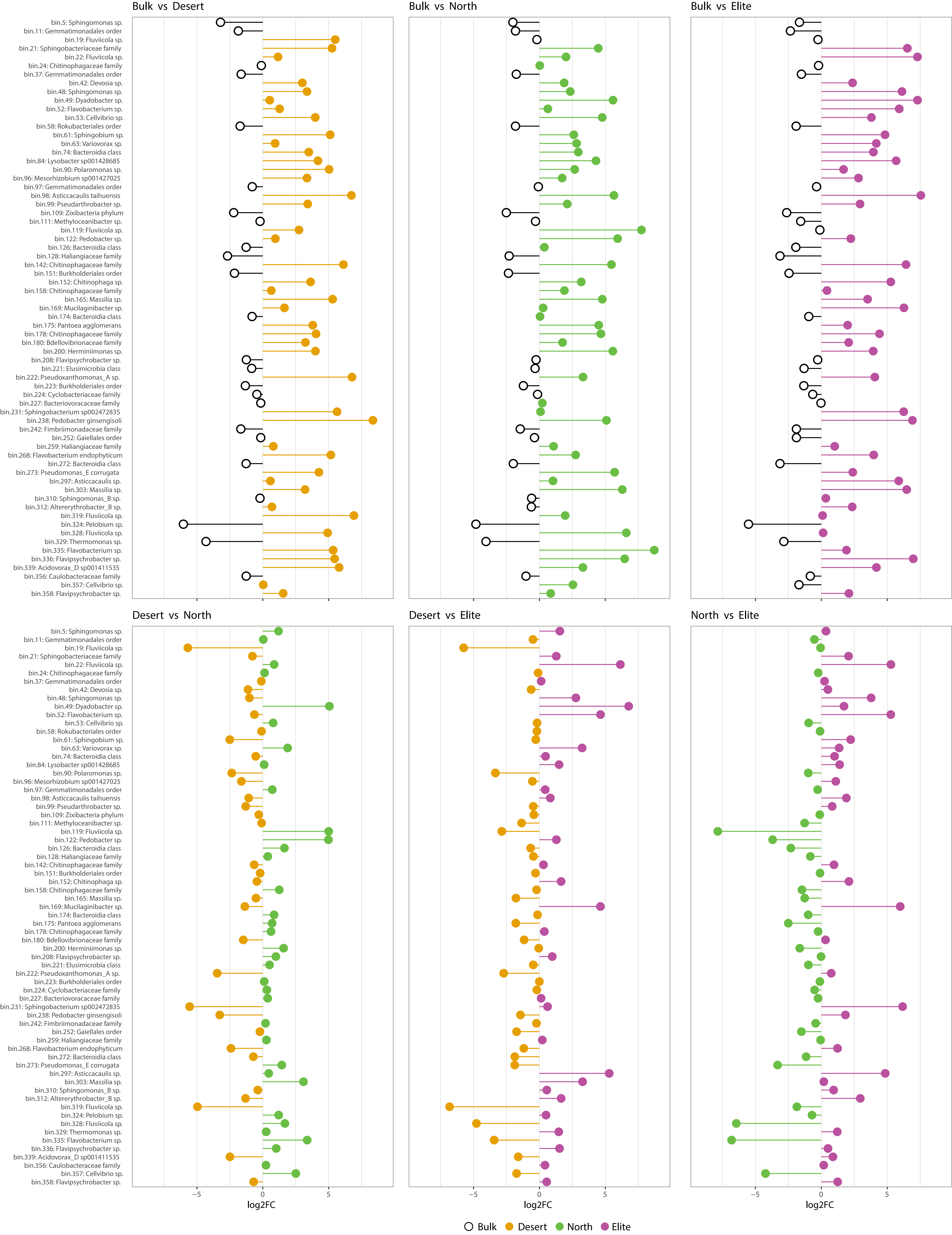
Bulk vs Desert
Bulk vs North
Bulk vs Elite
bin.5: Sphingomonas sp.
bin.11: Gemmatimonadales order
bin.19: Fluviicola sp.
bin.21: Sphingobacteriaceae family
bin.22: Fluviicola sp.
bin.24: Chitinophagaceae family
bin.37: Gemmatimonadales order
bin.42: Devosia sp.
bin.48: Sphingomonas sp.
bin.49: Dyadobacter sp.
bin.52: Flavobacterium sp.
bin.53: Cellvibrio sp.
bin.58: Rokubacteriales order
bin.61: Sphingobium sp.
bin.63: Variovorax sp.
bin.74: Bacteroidia class
bin.84: Lysobacter sp001428685
bin.90: Polaromonas sp.
bin.96: Mesorhizobium sp001427025
bin.97: Gemmatimonadales order
bin.98: Asticcacaulis taihuensis
bin.99: Pseudarthrobacter sp.
bin.109: Zixibacteria phylum
bin.111: Methyloceanibacter sp.
bin.119: Fluviicola sp.
bin.122: Pedobacter sp.
bin.126: Bacteroidia class
bin.128: Haliangiaceae family
bin.142: Chitinophagaceae family
bin.151: Burkholderiales order
bin.152: Chitinophaga sp.
bin.158: Chitinophagaceae family
bin.165: Massilia sp.
bin.169: Mucilaginibacter sp.
bin.174: Bacteroidia class
bin.175: Pantoea agglomerans
bin.178: Chitinophagaceae family
bin.180: Bdellovibrionaceae family
bin.200: Herminiimonas sp.
bin.208: Flavipsychrobacter sp.
bin.221: Elusimicrobia class
bin.222: Pseudoxanthomonas_A sp.
bin.223: Burkholderiales order
bin.224: Cyclobacteriaceae family
bin.227: Bacteriovoracaceae family
bin.231: Sphingobacterium sp002472835
bin.238: Pedobacter ginsengisoli
bin.242: Fimbriimonadaceae family
bin.252: Gaiellales order
bin.259: Haliangiaceae family
bin.268: Flavobacterium endophyticum
bin.272: Bacteroidia class
bin.273: Pseudomonas_E corrugata
bin.297: Asticcacaulis sp.
bin.303: Massilia sp.
bin.310: Sphingomonas_B sp.
bin.312: Altererythrobacter_B sp.
bin.319: Fluviicola sp.
bin.324: Pelobium sp.
bin.328: Fluviicola sp.
bin.329: Thermomonas sp.
bin.335: Flavobacterium sp.
bin.336: Flavipsychrobacter sp.
bin.339: Acidovorax_D sp001411535
bin.356: Caulobacteraceae family
bin.357: Cellvibrio sp.
bin.358: Flavipsychrobacter sp.
Desert vs North
Desert vs Elite
North vs Elite
bin.5: Sphingomonas sp.
bin.11: Gemmatimonadales order
bin.19: Fluviicola sp.
bin.21: Sphingobacteriaceae family
bin.22: Fluviicola sp.
bin.24: Chitinophagaceae family
bin.37: Gemmatimonadales order
bin.42: Devosia sp.
bin.48: Sphingomonas sp.
bin.49: Dyadobacter sp.
bin.52: Flavobacterium sp.
bin.53: Cellvibrio sp.
bin.58: Rokubacteriales order
bin.61: Sphingobium sp.
bin.63: Variovorax sp.
bin.74: Bacteroidia class
bin.84: Lysobacter sp001428685
bin.90: Polaromonas sp.
bin.96: Mesorhizobium sp001427025
bin.97: Gemmatimonadales order
bin.98: Asticcacaulis taihuensis
bin.99: Pseudarthrobacter sp.
bin.109: Zixibacteria phylum
bin.111: Methyloceanibacter sp.
bin.119: Fluviicola sp.
bin.122: Pedobacter sp.
bin.126: Bacteroidia class
bin.128: Haliangiaceae family
bin.142: Chitinophagaceae family
bin.151: Burkholderiales order
bin.152: Chitinophaga sp.
bin.158: Chitinophagaceae family
bin.165: Massilia sp.
bin.169: Mucilaginibacter sp.
bin.174: Bacteroidia class
bin.175: Pantoea agglomerans
bin.178: Chitinophagaceae family
bin.180: Bdellovibrionaceae family
bin.200: Herminiimonas sp.
bin.208: Flavipsychrobacter sp.
bin.221: Elusimicrobia class
bin.222: Pseudoxanthomonas_A sp.
bin.223: Burkholderiales order
bin.224: Cyclobacteriaceae family
bin.227: Bacteriovoracaceae family
bin.231: Sphingobacterium sp002472835
bin.238: Pedobacter ginsengisoli
bin.242: Fimbriimonadaceae family
bin.252: Gaiellales order
bin.259: Haliangiaceae family
bin.268: Flavobacterium endophyticum
bin.272: Bacteroidia class
bin.273: Pseudomonas_E corrugata
bin.297: Asticcacaulis sp.
bin.303: Massilia sp.
bin.310: Sphingomonas_B sp.
bin.312: Altererythrobacter_B sp.
bin.319: Fluviicola sp.
bin.324: Pelobium sp.
bin.328: Fluviicola sp.
bin.329: Thermomonas sp.
bin.335: Flavobacterium sp.
bin.336: Flavipsychrobacter sp.
bin.339: Acidovorax_D sp001411535
bin.356: Caulobacteraceae family
bin.357: Cellvibrio sp.
bin.358: Flavipsychrobacter sp.
−5
0
5
log2FC
−5
0
5
log2FC
−5
0
5
log2FC
Bulk
Desert
North
Elite
